# WGS Analysis of Clonal and Plasmidic Epidemiology of Colistin-Resistance Mediated by *mcr* Genes in the Poultry Sector in Lebanon

**DOI:** 10.3389/fmicb.2021.624194

**Published:** 2021-03-08

**Authors:** Hiba Al-Mir, Marwan Osman, Antoine Drapeau, Monzer Hamze, Jean-Yves Madec, Marisa Haenni

**Affiliations:** ^1^Laboratoire Microbiologie Santé et Environnement, Doctoral School of Sciences and Technology, Faculty of Public Health, Lebanese University, Tripoli, Lebanon; ^2^Université de Lyon – ANSES Laboratoire de Lyon, Unité Antibiorésistance et Virulence Bactériennes, Lyon, France

**Keywords:** *mcr-1*, poultry, Lebanon, IncX4, IncI2

## Abstract

Poultry and poultry meat are important contributors to the global antimicrobial burden. Unregulated and illegal use of extended-spectrum cephalosporins (ESC) in this sector has long been identified as a major cause of massive spread of ESC-resistant *Escherichia coli*, and colistin usage is considered a main driver of plasmid-mediated *mcr* genes dissemination. In Lebanon, the first *mcr-1*-positive *E. coli* found in poultry dates back to 2015, followed by a few reports of *mcr-1*-positive *E. coli* in poultry, swine, humans, and the environment. On the contrary, a comprehensive picture of the population structure of *mcr-1*-positive *E. coli* and *mcr-1*-bearing plasmids carrying the *mcr-1* gene using whole-genome analysis is largely lacking. This study reports the prevalence of *mcr-1*-positive *E. coli* in poultry originating from 32 farms across three Lebanese governorates and slaughtered in the same place. We report 27/32 (84.4%) *mcr-1* positive farms, leading to a total of 84 non-duplicate *E. coli* collected, of which 62 presented the *mcr-1* gene. Numerous associated resistances were identified, including to ESC through the presence of *bla*_CTX–M_ or *bla*_CMY_ genes. The *mcr-1* gene was mostly carried by IncX4 (*n* = 36) and IncI2 (*n* = 24) plasmids, which are both known for their efficient transfer capacities. A high genetic diversity was detected, arguing for the lack of contamination during the slaughter process. ST744 and ST1011 were the most widely identified clones, which have been both regularly associated to *mcr-1*-carrying *E. coli* and to the poultry sector. The wide dissemination of colistin-resistance, coupled to resistances to ESC and numerous other molecules, should urge authorities to implement efficient guidelines for the use of antibiotics in the poultry sector in Lebanon.

## Introduction

Since the discovery of the plasmid-mediated colistin-resistance gene *mcr-1* in 2015 (Liu et al., 2016), this gene has been extensively described in numerous animal settings, and notably in the poultry sector worldwide (Apostolakos and Piccirillo, 2018). Besides living animals, the *mcr-1* gene has also been detected in retail meat, suggesting a possible transfer to humans through under-cooked meat or cross-contamination (Nishino et al., 2017; Budel et al., 2020). To date, up to 10 *mcr* gene variants (*mcr-1* to *mcr-10*) have been recognized from different sources, of which *mcr-1* and *mcr-3* are the most widespread (Nang et al., 2019). Moreover, most studies have reported *mcr* genes in association with other resistance genes, including to critically important antimicrobials such as extended-spectrum cephalosporins (ESC) (Grami et al., 2016; Maciuca et al., 2019). In the poultry sector, *mcr-1* gene has mostly been found located on IncX4 and IncI2 plasmids, and to a lesser extent on IncHI2 plasmids (Perreten et al., 2016). The usually high transfer capacity of both IncX4 and IncI2 most probably explains their wide geographical dissemination and their occurrence in a large variety of hosts, both human and animals. Colistin-resistance has often been studied under the prism of plasmid-mediated resistance, so that only a few studies reported the characterization of *mcr*-negative but colistin-resistant isolates and the role of PmrAB and PhoPQ mutations (Quesada et al., 2015).

Regarding animals in Lebanon, *mcr-1*-mediated colistin-resistance was first reported in 2015 in poultry (Dandachi et al., 2018). A single *mcr-1*-positive *Escherichia coli* was recovered from one rectal swab over 982 samples (0.1%) taken in 49 farms for surveillance purposes. In 2017, a follow-up study was performed in this first *mcr*-1-positive farm, which showed that colistin-resistance had widely disseminated since 181/200 chicken and 6/6 workers carried *mcr-1*-producing *E. coli* or *Klebsiella pneumoniae*, while litter and feed were less heavily contaminated (6 and 20%, respectively) (Dandachi et al., 2020). In 2017 as well, 23 over 114 fecal samples from swine were resistant to colistin due to the presence of the *mcr-1* gene, and four of these isolates co-harbored resistances to ESC (Dandachi et al., 2019). Between 2017 and 2018, 88/93 (94.6%) fecal samples collected from the three major poultry farms in Lebanon presented the *mcr-1* gene, among which 35.5% co-harbored a *bla*_CTX–M_ gene (Hmede and Kassem, 2018). The presence of the *mcr-1* gene was also detected in water samples, from either irrigation water or the Mediterranean Sea (Hmede et al., 2019; Sourenian et al., 2020), suggesting environmental contamination. Finally, *mcr-1* has also been reported in human clinical isolates in Lebanon that had been collected as early as 2011 (Al-Mir et al., 2019). The plasmidic location of the *mcr-1* gene has, however, rarely been investigated in those studies: it was found located on an IncX4 plasmid in human isolates and in a rainbow trout isolate, and on IncX4 and IncI2 in water isolates (Al-Mir et al., 2019; Hassan et al., 2020; Sourenian et al., 2020). Likewise, data on the bacterial population structure hosting *mcr-1*-mediated colistin-resistance remain largely unknown.

The goal of this study was thus to look for the presence of colistin-resistance in chicken fecal samples collected from 32 chicken farms located in three governorates of Lebanon. Based on both phenotypic and molecular analyses (including next-generation sequencing), we characterized the population structure of colistin-resistant *E. coli*, the genetic support of *mcr*-dependent or *mcr*-independent colistin-resistance, and the plasmid types carrying the *mcr* genes in order to lay the foundations for a better understanding of colistin-resistance spread in poultry, but also in humans and the environment in Lebanon.

## Materials and Methods

### Ethics

This investigation was approved by the Azm Center/Lebanese University ethical committee (document CE-EDST-3-2018), authorized by the Lebanese Ministry of Public Health.

### Bacterial Isolation and Identification

**FIGURE 1:**
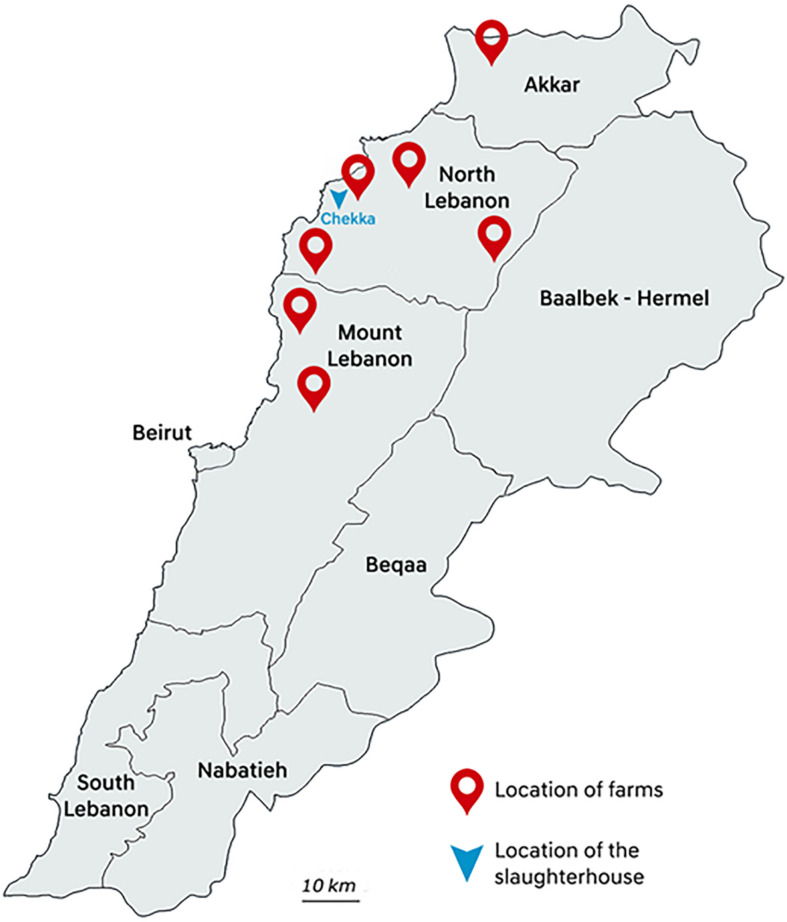
Map of the districts where sampling was performed.

Between May and August 2018, poultry samples were collected in one big slaughterhouse in Chekka, North Lebanon. Animals were originated from 32 individual farms hosting from 4500 to 195,000 chicken individuals, and located in seven districts from Akkar, North Lebanon, and Mount Lebanon governorates (Figure 1). At slaughterhouse, five different samples from each farm were collected: three caeca and two necks corresponding to five different chicken carcasses were randomly sampled with all precautions needed to avoid inter-sample contamination. Each farm was sampled once, except farm 2 that was sampled twice. All samples were put in plastic bags, conserved at 4°C, and rapidly transported to the Laboratoire Microbiologie Santé et Environnement (LMSE) in Tripoli, Lebanon. Resistant Enterobacterales were isolated on MacConkey agar (Bio-Rad, Hercules, CA, United States) supplemented with colistin (3.5 mg/L) (Sigma–Aldrich, St. Louis, MO, United States). Selective plates were incubated at 37°C for 24 h. One presumptive *E. coli* colony per morphology was arbitrarily selected from each selective plate. Identification of isolates was performed using matrix-assisted laser desorption/ionization time-of-flight MALDI TOF VITEK MS Version 3.0 (bioMérieux, Marcy L’Etoile, France).

### Antibiotic Susceptibility Testing and Phenotypic Characterization

Susceptibility testing was performed by the disc diffusion method on Mueller–Hinton agar according to the guidelines of the Antibiogram Committee of the French Society for Microbiology (CA-SFM)^1^. The *E. coli* ATCC 7624 strain was used as quality control. A total of 16 beta-lactam (amoxicillin, piperacillin, ticarcillin, amoxicillin-clavulanic acid, piperacillin-tazobactam, ticarcillin-clavulanic acid, cefalotine, cefuroxime, cefotaxime, ceftiofur, ceftazidime, cefoxitin, cefepime, cefquinome, aztreonam, and ertapenem) and 14 non-beta-lactam (tetracycline, kanamycin, tobramycin, gentamicin, amikacin, apramycin, netilmicin, streptomycin, florfenicol, chloramphenicol, sulfonamides, trimethoprim, nalidixic acid, and enrofloxacin) antibiotics of both veterinary and human interest were tested. Minimum inhibitory concentrations (MICs) were determined by broth microdilution for colistin, according to the European Committee for Antimicrobial Susceptibility Testing (EUCAST).

### Molecular Typing of the Isolates

The detection of the major *E. coli* phylogenetic groups (A, B1, B2, or D) was performed as described by Doumith et al. (2012). Duplicate isolates collected from the same farm were detected by multiple-locus variable-number tandem-repeat analysis (MLVA) using the multiplex-based PCR described by Caméléna et al. (2019).

### Whole-Genome Sequencing (WGS)

DNA was extracted using the NucleoSpin Microbial DNA extraction kit (Macherey-Nagel, Hoerdt, France) according to the manufacturer’s instructions. Library preparation was performed using the Nextera XT technology, and sequencing was performed on a NovaSeq instrument (Illumina, San Diego, CA, United States). After sequencing, reads were quality trimmed and *de novo* assembled using Shovill v1.0.4, and the quality of assemblies was assessed using QUAST v5.0.2. STs were determined using the CGE online tool MLSTFinder v2.0.4^2^, while resistance genes and replicon content were inferred using ABRicate v1.0.1^3^. Mutations in *gyrA* and *parC* were searched using PointFinder (see text footnote 2). The PmrA, PmrB, PhoP, and PhoQ amino acid sequences were extracted from the assemblies of all isolates and compared using Clustal Omega with the *E. coli* K12 reference strain (NP_418537.1 for PmrA, NP_418536.1 for PmrB, NP_415648.1 for PhoP, and NP_415647.1 for PhoQ). Virulence factors (VFs) were determined using VirulenceFinder, and serotypes were determined using SeroType Finder (see text footnote 2). Avian pathogenic *E. coli* (APEC) was defined according to Johnson et al. (2008).

### Characterization of the *mcr-1*-Carrying Plasmids

The replicon content was determined from the whole-genome sequencing (WGS) data using PlasmidFinder 2.0.1 (see text footnote 2). Plasmids carrying the *mcr-1* gene were assigned *in silico* when the *mcr-1* gene was located on the same contig as the plasmidic marker. When *in silico* data showed no co-occurrence on the same contig, plasmids carrying the *mcr-1* gene were detected using PFGE-S1 gels (6 V/cm for 20 h with an angle of 120° at 14°C with pulse times ranging from 1 to 30 s) followed by Southern blot using adequate probes as previously described (Saidani et al., 2019). Plasmid co-localization was assessed by comparison between the bands corresponding to the resistance gene and those corresponding to the Inc type of the plasmid. When Southern blots did not lead to interpretable results and for all isolates that could not be typed by PFGE (smearing profile), the plasmid of interest was transferred by conjugation in an *E. coli* J53 rifampicin-resistant recipient strain. Conjugation was performed in liquid medium using rifampicin and cefotaxime (5 mg/L) or colistin (2 mg/L) to select transconjugants (TC). Only TC presenting a unique plasmid, as assessed by S1-PFGE, were further characterized by PBRT and Southern blots as described above.

### Phylogenetic Analysis

The core genome multi-locus sequence type (cgMLST) was extracted from the WGS data. The pan-genome was determined, and core gene alignments were generated, for each collection, with Roary v. 3.13.0 (Page et al., 2015) using a Protein BLAST identity of 80% and a core definition of 90%. In the first step, all assemblies were annotated *de novo* with Prokka v1.14.6 using default settings (Seemann, 2014). The Prokka annotations were provided to Roary as input. Subsequently, recombination was removed with gubbins v2.4.1 and a maximum likelihood tree was constructed from the core gene alignment produced by Roary using RAxML v.8.2.12 using default parameters. Pairwise single nucleotide polymorphism (SNP) distances were calculated from core genome alignments generated by Roary using snp-dists^4^. The SNP distance matrix is provided as Supplementary Table S1. The resulting tree for both analysis/collections was visualized using iTol v.5.5.1^5^ (Letunic and Bork, 2019).

### Statistical Analyses

Statistical analyses were performed with GraphPad Prism 6.0 (GraphPad Software, Inc., San Diego, CA, United States) using chi-square test in order to search for an association between *mcr-1* gene and ESBL or AmpC genes among colistin-resistant isolates. The tests were two-sided, with a type I error set at α = 0.05.

### Accession Number(s)

The whole genome shotgun project was deposited in DDBJ/EMBL/GenBank under the BioProject accession number PRJNA671785.

## Results

### Detection of Colistin-Resistance

All presumptive colistin-resistant *E. coli* isolated on selective plates was characterized according to their resistance phenotype, phylogroup, and MLVA profiles, and only non-duplicate isolates were kept for further studies. Among the 32 farms tested, 27 presented at least one colistin-resistant isolate (27/32, 84.4%), with MICs ranging from 4 to 64 mg/L. Since several different isolates were retrieved from one farm (up to 12), and also from one animal (up to three), a total of 84 colistin-resistant isolates were collected (Supplementary Table S1).

### *E. coli* Characterization and Virulence Patterns

The 84 *E. coli* isolates belonged to phylogroups A (*n* = 44, 52.4%), B1 (*n* = 22, 26.2%), B2 (*n* = 1, 1.2%), and D (*n* = 17, 20.2%). Thirty-one different serogroups were identified, but only O21 (*n* = 14), O102 (*n* = 8), O101 (*n* = 6), O38 (*n* = 5), O1 (*n* = 3), and O109 (*n* = 3) were found in more than two isolates. Twenty-four isolates (24/84, 28.6%) could be considered as APEC according to the definition by Johnson et al. (2008), i.e., concomitantly presenting the *iss*, *iutA*, *hlyF*, *iroN*, and *ompT* virulence genes (Supplementary Table S1).

### Plasmid-Mediated Colistin-Resistance

Whole-genome sequencing data revealed that no other *mcr* gene than *mcr-1* was present in the *E. coli* genomes. The *mcr-1* gene was detected in 62 *E. coli* isolates (62/84, 73.8%) originating from 21 different farms. Two copies of *mcr-1* were found in one isolate, located on the same contig. The plasmidic location of the *mcr-1* gene was proved in 61/62 of the isolates, mostly by deduction from WGS data when *mcr-1* and Inc genes were located on the same contig (37 isolates) or next to conjugation and Southern blot experiments (14 isolates). For one isolate, the plasmidic or chromosomal location of *mcr-1* was not resolved despite numerous attempts (absence of conjugation and smearing PFGE profile). In all, the *mcr-1* gene was mostly carried by an IncX4 plasmid (*n* = 36), followed by an IncI2 (*n* = 24) and IncHI2 (*n* = 1) plasmid.

### Chromosome-Mediated Colistin-Resistance

Amino-acid variations in the PhoP, PhoQ, PmrA, and PmrB were extracted from the WGS data of all *mcr-1*-positive and *mcr-1*-negative isolates (Supplementary Table S1 and Table 1). Modifications were found at one site in PhoP, 11 sites in PhoQ, two sites in PmrA, and six sites in PmrB. Four modifications (H2R and D283G in PmrB, I44L in PhoP, and A482T in PhoQ) were found in ≥25 isolates. Eleven modifications (one in PmrA and 10 in PhoQ) were found only in *mcr-1*-positive isolates (Table 1), while two modifications in PhoQ (three isolates with the V228I modification and one isolate with the T348I modification) were exclusively found in *mcr-1*-negative isolates. However, no pattern and not even one specific mutation were found to be associated with all 22 *mcr-1*-negative colistin-resistant isolates.

**TABLE 1:** Amino-acid modifications (PmrA, PmrB, PhoP, and PhoQ) in *mcr-1*-positive and *mcr-1*-negative isolates.

	PmrA		PmrB						PhoP	PhoQ											
	G29S	V129L	H2R	A242V	D283G	V351F	Y358N	A360V	I44L	I175F	V228I	A341T	N346K	T348I	T348N	V373I	V386L	A390T	E464D	L467M	A482T
*mcr-1*-positive isolates	1	2	61	15	27	15	11	2	21	2	0	1	1	0	2	1	1	1	19	2	30
*mcr-1*-negative isolates	1	0	21	1	17	2	7	2	4	0	3	0	0	1	0	0	0	0	0	0	0
Total	2	2	82	16	44	17	18	4	25	2	3	1	1	1	2	1	1	1	19	2	30

### Associated Resistance Genes

**FIGURE 2:**
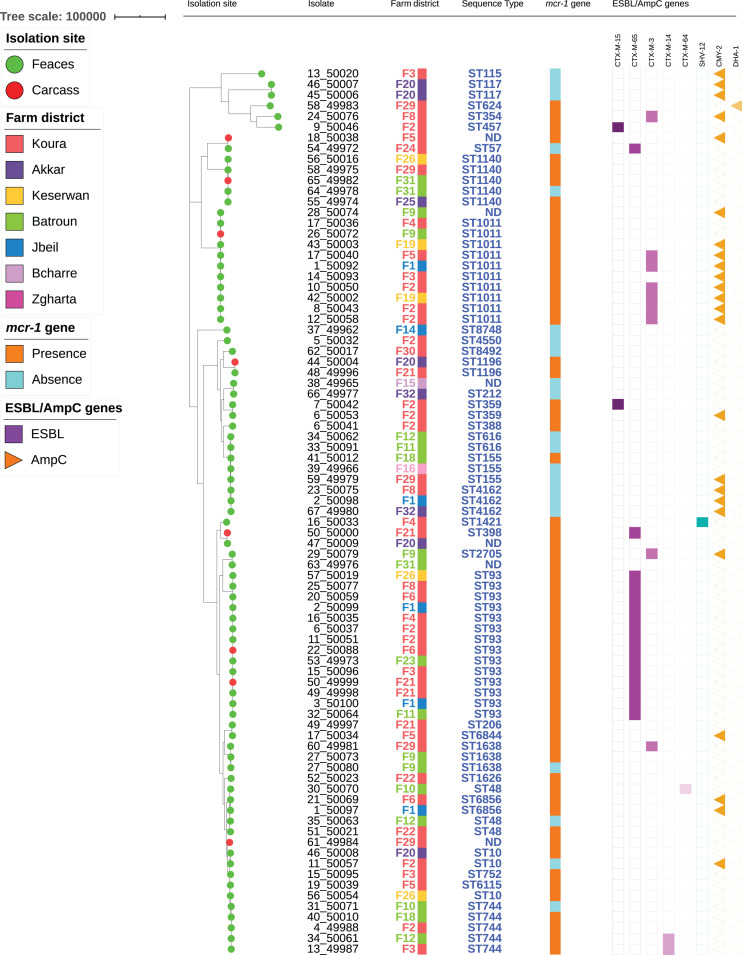
Maximum-likelihood phylogeny of *E. coli* isolates from poultry. The phylogenetic tree was constructed based on nucleotide sequence alignments of the core genes. Metadata columns include farm district, STs, and presence/absence of the *mcr-1* gene and resistance genes.

Among the 62 *mcr-1*-positive *E. coli* isolates, 30 presented an associated ESBL gene, including *bla*_CTX–M–__65_ (*n* = 15), *bla*_CTX–M–__3_ (*n* = 9), *bla*_CTX–M–__14_ (*n* = 2), *bla*_CTX–M–__15_ (*n* = 2), *bla*_CTX–M–__64_ (*n* = 1), and *bla*_SHV–__12_ (*n* = 1) (Figure 2). No ESBL/*mcr-1* co-localization could be evidenced, neither by conjugation nor by analyzing WGS data. Plasmidic AmpC genes were also detected in 17 isolates, with *bla*_CMY–__2_ in 16 of them. All but one CTX-M-3-positive *E. coli* isolate also displayed the CMY-2 enzyme. On the contrary, only one CTX-M-65 and nine CMY-2-producing *E. coli* were identified among *mcr-1*-negative colistin-resistant isolates. The presence of the ESBL genotype was 20-fold more common in *mcr-1*-positive than in *mcr-1*-negative colistin-resistant isolates (OR: 19.7, CI: 2.5–155.6, *P* = 0.0003). On the contrary, no significant difference related to AmpC phenotype was observed between the two aforementioned colistin-resistant groups.

In addition to genes responsible for colistin-resistance, *E. coli* isolates mostly presented resistance genes to aminoglycosides (75/84, 89.3%), tetracyclines (71/84, 84.5%), sulfonamides (66/84, 78.6%), trimethoprim (62/84, 73.8%) and phenicols (53/84, 63.1%). The corresponding genes were mainly *tet*(A), *sul2*, *dfrA12*, and *floR*, while aminoglycoside resistance was largely multifactorial (Supplementary Table S1). Quinolone-resistance was observed in 70 isolates, of which 56 were also resistant to fluoroquinolones. Variants of the plasmid-mediated resistance gene *qnrB* (*n* = 4), as well as the *qnrS1* (*n* = 14) gene were detected in 14 different isolates. The mutations S83L and D87N in GyrA and S80I in ParC were concomitantly detected in 47/56 fluoroquinolone-resistance isolates (Supplementary Table S1). Only one apramycin-resistant isolate was identified, which presented the *aac*(3′)-IV gene, while none of the isolates was resistant to ertapenem and amikacin (Table 2).

**TABLE 2:** Associated resistance phenotypes.

	*mcr-1-*positive isolates (*n* = 62)		*mcr-1*-negative isolates (*n* = 22)		Total (*n* = 84)	
	No.	Percentage	No.	Percentage	No.	Percentage
Streptomycin	31	50.0	17	77.3	48.0	57.1
Kanamycin	44	71.0	15	68.2	59.0	70.2
Amikacin	0	0.0	0	0.0	0.0	0.0
Apramycin	1	1.6	0	0.0	1.0	1.2
Gentamicin	26	41.9	9	40.9	35.0	41.7
Tobramycin	24	38.7	9	40.9	33.0	39.3
Netilmicin	11	17.7	6	27.3	17.0	20.2
Chloramphenicol	42	67.7	11	50.0	53.0	63.1
Florfenicol	35	56.5	9	40.9	44.0	52.4
Tetracyclines	51	82.3	17	77.3	68.0	81.0
Sulfonamides	46	74.2	18	81.8	64.0	76.2
Trimethoprim	47	75.8	17	77.3	64.0	76.2
Nalidixic acid	53	85.5	17	77.3	70.0	83.3
Enrofloxacin	47	75.8	9	40.9	56.0	66.7

### Population Structure of the *mcr-1*-Positive and *mcr-1*-Negative *E. coli* Isolates

The 84 isolates belonged to 38 different STs (Figure 2 and Supplementary Table S1). Two STs were predominant [ST93 (*n* = 14) and ST1011 (*n* = 11, including one SLV)], while two were each found in five different isolates (ST744 and ST1140). The remaining isolates were identified in three or less isolates. Within one ST, colistin-resistance was usually mediated by the same mechanism (either *mcr-1* or chromosomal mutations). However, six STs (ST10, ST48, ST155, ST744, ST1140, and ST1635) encompassed isolates that were resistant to colistin through either plasmid- or chromosome-mediated mechanisms.

Among the STs presenting five isolates or more, only ST93 formed a homogeneous group, with isolates differing by 14–72 SNPs. On the other side, ST744, ST1011, and ST1140 were split into two groups, each differing by > 900, > 2700 SNPs, and > 600 SNPs, respectively (Supplementary Table S2). The number of SNPs was also detailed for all isolates sharing the same ST. ST359 presented two highly similar isolates (25 SNPs) originating from two different animals from the same farm, while ST616 and ST1196 each comprised two isolates from different farms that only differed by 3 SNPs. On the contrary, ST48 and ST6856 each included two isolates from different farms, respectively, differing by > 5000 and 391 SNPs. ST4162 comprised three isolates from different farms, of which two (farms F1 and F32) were fully identical, while the third one (F8) differed by 27 SNPs. ST155 encompassed three isolates from three different farms: isolates from farms F16 and F29 displayed the *mcr-1* gene and differed by 34 SNPs, while the isolate from farm F18 was *mcr-1*-negative and differed from the two others by > 5000 SNPs. ST1638 comprised three isolates: two were from the same farm F9 and were highly similar (5 SNPs), while the third one from farm F22 clearly diverged (>5000 SNPs). Finally, ST10 included three isolates from three different farms that were not similar, isolates from farm F2 and F20 differing by > 200 SNPs, and further differing by > 6000 SNPs from the isolate from farm F26.

## Discussion

This study first reveals a massive spread of colistin-resistance in poultry farms in Lebanon (27/32, 84.4%), with the wide dissemination of the *mcr-1* gene (21/27 positive farms). The poultry sector is the very first one where *mcr-1* was detected in Lebanon (Dandachi et al., 2018), and a recent study in three poultry farms reported high *mcr-1* prevalence in broilers (Hmede and Kassem, 2018). The present work on 32 farms therefore expands our knowledge on the magnitude of *mcr-1* spread in the poultry sector in Lebanon. This high prevalence can most probably be explained by the unregulated use of colistin in this country in the poultry sector, without implementation of antibiotic stewardship programs (Kassem et al., 2019). Even if we have no specific information on colistin use in the farms from which our samples originated, it is known that colistin is easily available without the requirement of a veterinarian’s prescription. Around 12 different drug brands that contain colistin are legally available over the counter in agriculture stores in Lebanon and particularly advised for the treatment and prevention of infections in poultry. Consequently, there is a high probability that colistin had been used in several of the tested farms. Additional ESBL/pAmpC genes were detected in 49/84 *E. coli* isolates, and ESBL genes were statistically more associated with *mcr-1*-positive isolates. These 49 ESBL/pAmpC-positive isolates, as well as 28 of the 35 remaining isolates, were multidrug-resistant (resistance to three or more antibiotic families). This suggests that colistin-resistance would be easily co-selected in the poultry gut by the use of most of the veterinary-licensed antibiotics. The *bla*_ESBL_ genes found in these isolates (*bla*_CTX–M–__3_ and *bla*_CTX–M–__65_) do not betray a human origin, where the *bla*_CTX–M–__15_ gene (which was only detected in two poultry isolates) is clearly dominating.

The mechanism of colistin-resistance in *mcr*-negative isolates was not elucidated here. Numerous mutations were observed compared to the K12 reference strain, most of which were detected in both *mcr*-positive and *mcr*-negative isolates, if not only in *mcr*-positive ones. The rare mutations that have been associated with colistin-resistance (R81S in PmrA; T156K, A159V, G161V in PhoP; E375K in PhoQ) were not detected in our dataset (Jeannot et al., 2017). Likewise, the N346K modification highlighted by Luo et al. (2017) as a possible colistin-resistance-related modification was found here associated to *mcr-1*-positive isolates. We might hypothesize that colistin-resistance-related mutations in PmrAB and PhoPQ may also arise in *mcr-1*-positive isolates and contribute to an increased MIC to colistin, but the highest MICs to colistin (≥16mg/L) observed in this study did not correlate with a specific modification pattern. Moreover, several modifications detected here (S29G in PmrA, H2R in PmrB, and D283G in PhoQ) have also been described in susceptible isolates (Luo et al., 2017). Consequently, further studies are needed to identify other genes that may be associated to colistin-resistance.

The *E. coli* population structure described here appeared very diverse, with 38 different STs detected, as it is mostly the case when samples are originating from different farms. Since all animals were sampled in the same slaughterhouse (even though poultry originated from 32 different farms), we looked for a potential contamination related to the slaughterhouse. Such a massive contamination is very unlikely because collected *E. coli* were genetically diverse, which rather indicates multiple origins. Nevertheless, a one-source contamination cannot be excluded in the cases of ST616 and ST1196 (two isolates each, originating from different farms) and ST93 (recovered in 14 isolates collected from 10 different farms), which only differed by a few SNPs. Clonal spread of *mcr-1*-carrying ST93 clinical isolates has been described in companion animals attending a veterinary hospital in China (Wang et al., 2018), suggesting that this clone may survive in the environment (a clinic or a slaughterhouse) before further dissemination. Interestingly, the *mcr-1* gene has also been detected in ST93 *E. coli* isolated from a human patient in Uruguay and from one healthy person in Finland (Grondahl-Yli-Hannuksela et al., 2018; Papa-Ezdra et al., 2020).

Besides ST93, and despite the high clonal diversity, two other main STs (ST744 and ST1011) were detected. These STs have already been both concomitantly reported in *mcr-1*-positive isolates of poultry origin in Czechia and in colistin-susceptible poultry isolates from Algeria (Belmahdi et al., 2016; Gelbicova et al., 2019). ST1011 has also been reported in *mcr-1*-positive environmental samples of swine farms in Germany, in pigs in China and Belgium, in poultry in Egypt, and in companion animals in China, as well as in colistin-susceptible poultry meat isolates in Egypt (Elnahriry et al., 2016; El Garch et al., 2017; Guenther et al., 2017; Wang et al., 2018; Ramadan et al., 2020; Shen et al., 2020). Interestingly, *mcr-1*-positive ST1011 *E. coli* isolates have also been recently identified from poultry farm workers in Lebanon (Dandachi et al., 2020). Consequently, the *mcr-1*-positive ST1011 *E. coli* isolate that was identified in 2013 in a Lebanese patient may well have a poultry (or at least an animal) origin (Al-Mir et al., 2019). ST744 has also been reported in *mcr-1*-positive isolates from poultry in Romania and from swine in China, and from *mcr-3*-producing *E. coli* in veal calves in France (Haenni et al., 2018; Maciuca et al., 2019; Shen et al., 2020), but also from *mcr-1*-positive clinical *E. coli* in Portugal (Tacao et al., 2017), suggesting that this clone may be particularly prone to harbor colistin-resistance. Nonetheless, it should be kept in mind that WGS data nowadays strongly challenge any lineage distribution based on MLST only, as also highlighted in some occasions in the present work. Therefore, WGS approaches are required for any further comprehensive pictures of the cross-sectorial distribution of *mcr-1*-positive *E. coli*.

A strength of our work refers to the identification of *mcr-1*-bearing plasmids, which was almost absent from other studies in Lebanon (Al-Mir et al., 2019; Hassan et al., 2020; Sourenian et al., 2020). With the single exception of one isolate where plasmid or chromosomal location was not clarified, all *mcr-1* genes were detected on plasmids, mostly on IncX4 (*n* = 36) but also on IncI2 (*n* = 24). IncX4 is the main plasmid spreading *mcr* genes worldwide, and notably *mcr-1* (Matamoros et al., 2017). IncX is a family of small and narrow-range plasmids, and experiments proved that it has a very weak fitness cost and high transfer frequencies at 30°C, allowing its wide spread in environmental settings (Lo et al., 2014). IncI2 plasmids are also spreading efficiently globally, and a recent study proved *in situ* in a mouse model that this plasmid family had a particularly high capacity to transfer DNA in the gut (Neil et al., 2020). This high transfer capacity of both IncX4 and IncI2 plasmids may explain the occurrence of *mcr-1* in such a high number of genetic backgrounds of *E. coli*, and the relative absence of clonal transmission on farm, since animals from the same farm mostly carried different *E. coli* clones.

## Conclusion

We report a high prevalence and a massive spread of *mcr-1*-positive *E. coli* in poultry farms in Lebanon. Based on WGS analysis, we deciphered that the colistin-resistance gene *mcr-1* has widely disseminated in the poultry sector in diverse genetic backgrounds of *E. coli*, and principally on IncX4 and IncI2 plasmids. Interestingly, no other *mcr* variant than *mcr-1* was found in this sector, while other still unknown non-*mcr* genes most likely also contribute to colistin-resistance. Also, a comprehensive WGS-based picture of the global clonal and plasmidic epidemiology of *mcr-1*-positive *E. coli* in a One Health perspective still lacks in all sectors to further conclude or hypothesize on major sources and routes of transmission in Lebanon. Nonetheless, some situations would warrant further investigations, such as the occurrence of ST1011 in poultry (where it is a major *E. coli* clone), in a human patient and a poultry farm worker in the same country. These results should be used to inform and increase breeders’ awareness of the consequences of uncontrolled use of antibiotics in their daily practices. Overall, the wide dissemination of colistin-resistance, coupled to a low-level awareness of antibiotic stewardship in the Lebanese community (Al Omari et al., 2019) and high resistance rates to ESC and numerous other molecules, should urge authorities to implement efficient guidelines for the use of antibiotics in the poultry—and more globally the Agri-food sector in Lebanon.

## Data Availability Statement

The datasets presented in this study can be found in online repositories. The names of the repository/repositories and accession number(s) can be found in the article/Supplementary Material.

## Ethics Statement

The animal study was reviewed and approved by the Azm Center/Lebanese University Ethical Committee (document CE-EDST-3-2018), authorized by the Lebanese Ministry of Public Health.

## Author Contributions

MH and MO designed the experiments and supervised the sampling campaign. HA-M performed the experiments. AD performed all WGS analyses. MH, HA-M, and J-YM analyzed the data. MH drafted the manuscript. All authors approved the final version of this manuscript.

## Conflict of Interest

The authors declare that the research was conducted in the absence of any commercial or financial relationships that could be construed as a potential conflict of interest.

## Footnotes

1 www.sfm-microbiologie.org

2 http://www.genomicepidemiology.org/

3 https://github.com/tseemann/abricate

4 https://github.com/tseemann/snp-dists

5 http://itol.embl.de/itol.cgi
